# Development and validation of a predictive model for invasive syndrome in patients with *Klebsiella pneumoniae* liver abscess

**DOI:** 10.3389/fmed.2025.1663407

**Published:** 2025-09-18

**Authors:** Liyong Zhang, Jiaqi Chen, Yihao Qu, Xidong Cao, Jinhua Cui, Jian Li, Aijun Yu

**Affiliations:** The First Department of General Surgery, Affiliated Hospital of Chengde Medical University, Chengde, China

**Keywords:** *Klebsiella pneumoniae*, liver abscess, invasive syndrome, predictive model, nomogram

## Abstract

**Introduction:**

Pyogenic liver abscess (PLA) is a life—threatening liver bacterial infection causing suppurative lesions. In Asia, hypervirulent *Klebsiella pneumoniae* (hvKP) is the main PLA pathogen, linked to invasive syndromes. Severe *Klebsiella pneumoniae* liver abscess (KPLA) manifestations, called invasive KPLA syndrome (IKPLAS), have acute onset, rapid progression and non-specific symptoms, often leading to poor prognosis if untreated. This study aimed to find risk factors and create a validated nomogram for predicting invasive syndrome in KPLA patients.

**Methods:**

We retrospectively analyzed clinical data from 529 KPLA patients treated at Chengde Medical University Affiliated Hospital between August 1, 2014, and November 30, 2024. By using the 7:3 stratified random sampling method, the patients were assigned to two cohorts: derivation (*n* = 370) and validation (*n* = 159). Univariate and multivariate logistic regression analyses were performed to identify risk factors for invasive KPLA syndrome (IKPLAS). A predictive nomogram was constructed and evaluated for discrimination and clinical utility.

**Results:**

Of the 529 enrolled patients, 33 patients (6.2%) developed IKPLAS (IKPLAS group), while the remaining 496 patients were included in the non-invasive group (NIKPLAS group). Both groups showed significant differences (*P* < 0.05) in the incidence of viral hepatitis, biliary disease, type 2 diabetes mellitus (T2DM), vomiting, pulmonary infection, and septic shock; C-reactive protein level; abscess diameter; presence of a gas-containing abscess; and Sequential Organ Failure Assessment (SOFA) score. Multivariate analysis identified the following factors as independent predictors: viral hepatitis, T2DM, abscess diameter, presence of a gas-containing abscess, and SOFA score. The nomogram showed excellent calibration (Hosmer–Lemeshow χ^2^ = 4.171, *P* = 0.841) with area under the receiver operating characteristic curve values of 0.961 (derivation cohort) and 0.899 (validation cohort). The clinical utility of the nomogram was confirmed by decision curve analysis.

**Conclusion:**

Viral hepatitis, T2DM, abscess diameter, presence of a gas-containing abscess, and SOFA score are the predictive factors of IKPLAS. The developed nomogram provides reliable risk stratification for patients with KPLA and can be applied clinically to predict IKPLAS cases.

## 1 Introduction

Pyogenic liver abscess (PLA) is a life-threatening bacterial infection of the liver that leads to suppurative lesions (1, 2), and it has emerged as a global public health concern, particularly in developing countries with an increasing incidence of PLA (3). A comprehensive understanding of the risk factors for PLA is crucial for the early identification of high-risk populations and the prevention of disease progression. The key risk factors associated with the development of PLA can be categorized into the following groups: systemic underlying diseases, such as diabetes mellitus, malignancy, and severe immunodeficiency disorders; biliary system abnormalities, including cholangitis, cholelithiasis (gallstones), and biliary strictures—these conditions facilitate the ascending spread of bacteria from the biliary system to the liver; gastrointestinal tract-related factors, such as appendicitis, diverticulitis, and inflammatory bowel disease, where intestinal bacteria can enter the liver via the portal venous circulation; and iatrogenic factors, which include percutaneous transhepatic biliary drainage, endoscopic retrograde cholangiopancreatography (ERCP), and liver surgery—pathogens may be introduced during these invasive procedures (4).While bacteria are the most common pathogens of liver abscesses, PLA can also be caused by other microorganisms besides bacteria. Specifically, certain fungi (such as Candida species, including Candida albicans and Candida glabrata) and a small number of protozoa (e.g., *Entamoeba histolytica*) have been identified as pathogenic microorganisms responsible for PLA.

In Asia, hypervirulent *Klebsiella pneumoniae* (hvKP) is recognized as the predominant pathogen of PLA and is strongly associated with the development of invasive syndromes such as endophthalmitis, meningitis, and necrotizing fasciitis (5). Notably, the global incidence of *Klebsiella pneumoniae*-associated liver abscess (KPLA) has increased substantially, with 15%−25% of KPLA cases progressing to extrahepatic metastatic infections (6). These severe manifestations of KPLA, collectively termed invasive *Klebsiella pneumoniae* liver abscess syndrome (IKPLAS), are characterized by acute onset, rapid progression, and non-specific clinical presentation, frequently resulting in poor prognosis and mortality if not promptly diagnosed and treated (7). It is important to note that while metastatic infections have been considered as an inclusion criterion for IKPLAS in the present study, existing literature also reports cases of invasive *Klebsiella pneumoniae* pyogenic liver abscess without evidence of metastatic infection. Such cases typically exhibit aggressive local progression (e.g., extensive hepatic parenchymal necrosis or biliary obstruction due to abscess compression) or severe systemic inflammation (such as persistent high fever, septic shock, or multiple organ dysfunction syndrome), which also fulfill the clinical definition of “invasive” disease due to their life-threatening nature. Recent research studies have progressively elucidated the pathogenic mechanisms of hvKP-associated liver abscesses, which involve synergistic interactions among capsular serotypes (K1/K2), virulence plasmids, and host factors (8). Current risk prediction models focus predominantly on single-dimensional biomarkers, failing to incorporate the complexity of multifactorial interactions to deliver optimal outcomes (9). Moreover, conventional scoring systems show limited specificity for diagnosing invasive syndromes, thereby restricting their clinical application (10). The lack of widely validated predictive tools for the early identification of high-risk populations leads to delayed clinical interventions, highlighting the critical need to develop multidimensional risk stratification models.

Clinical prediction models are multivariate statistical tools designed to integrate prognostic indicators for risk stratification (11). Previous predictive models for the risk factors of IKPLAS have mostly focused on correlation analysis and exploration of the underlying mechanisms (12). Although the key risk factors are known, systematic quantitative assessment tools are lacking. Hence, in the present study, we constructed a simple and intuitive nomogram by elucidating the independent risk factors influencing the development of the invasive syndrome in patients with KPLA. This assessment tool integrates complex multidimensional risk factors with intuitive quantitative indicators, which can substantially improve the accuracy of risk prediction and provide a more scientific basis for clinical decision-making. This model can also enable hierarchical management of the risks of individuals or groups, facilitate efficient allocation of resources, and assist high-risk populations in prioritizing access to targeted preventive interventions.

## 2 Materials and methods

### 2.1 Study population

This retrospective study analyzed electronic medical records of patients diagnosed to have liver abscess at Chengde Medical University Affiliated Hospital between August 1, 2014, and November 30, 2024. This study was approved by the Ethics Committee of the Affiliated Hospital of Chengde Medical University (approval number: CYFYLL2022507). The requirement for informed consent was waived because of the retrospective nature of this study. Two investigators independently reviewed the case records according to the following predefined PLA diagnostic criteria: (1) clinical symptoms, including fever, vomiting, jaundice, and upper abdominal pain; (2) radiologic confirmation of hepatic abscess/lesions through ultrasonography/CT/MRI; (3) positive blood or abscess culture or therapeutic response to antibiotics; and (4) definitive diagnosis through percutaneous/surgical drainage. To confirm PLA diagnosis, criteria (1) and (2) and more than 1 item from criteria (3) and (4) were required to be met. The inclusion criteria for IKPLAS were as follows: (1) confirmed PLA diagnosis; (2) positive *Klebsiella pneumoniae* culture (blood/abscess); and (3) invasive infection (e.g., endophthalmitis or meningitis). The exclusion criteria were as follows: (1) age < 18 years; (2) primary diagnosis is not PLA; and (3) missing clinical and imaging data.

### 2.2 Data collection

The following demographic and clinical data were extracted from the electronic records: (1) general information: age and gender; (2) comorbidities: viral hepatitis, biliary disease, malignant neoplasm, hypertension, T2DM, and hyperuricemia; (3) clinical features: temperature, shiver, poor appetite, vomiting, abdominal pain, pleural effusion, ascites, pulmonary infection, perihilar abscess, septic shock, and multiple organ dysfunction syndrome (MODS); (4) laboratory parameters: white blood cell (WBC) count, neutrophil ratio, platelet count (PLT), hemoglobin (Hb) level, c-reactive protein (CRP) level, blood glucose level, alkaline phosphatase (ALP) level, international normalized ratio, and fibrinogen level; (5) imaging findings: abscess location, number of abscesses, abscess diameter, and presence of a gas-containing abscess; (6) treatments: antibiotic treatment, antibiotics and drainage, and operative drainage. The Sequential Organ Failure Assessment (SOFA) score was also calculated. Baseline laboratory results alone were included in the analysis.

### 2.3 Statistical analysis

SPSS (version 26.0) and R software (version 4.4.2) were used to perform statistical analysis. The cohort data were categorized into a derivation cohort (*n* = 370) and a validation cohort (*n* = 159). Variables with more than 20% missing values (procalcitonin, interleukin, and lactic acid levels) were excluded from the analysis. For variables with the missingness rate of < 20% (fibrinogen, CRP, and ALP levels), the missing data were compensated using the fully conditional specification multiple imputation method. Ten imputation cycles were performed, with algorithm specifications tailored according to the variable types: predictive mean matching for continuous variables, logistic regression analysis for binary categorical variables, and proportional odds model for ordinal categorical variables. Continuous variables were compared among the groups by using *t*-test or Wilcoxon rank-sum test. Categorical variables are presented as frequencies (percentages), and continuous variables are expressed as mean ± standard deviation (SD) or median (P25, P75). Initially, univariate analysis was conducted to identify factors associated with invasive syndrome in patients with liver abscesses. Statistically significant factors (*P* < 0.05) from the univariate analysis were included in the multivariate analysis. Multivariate logistic regression analysis with bidirectional stepwise selection was conducted to identify independent clinical predictors. Inclusion and exclusion probability thresholds of *P* < 0.05 and *P* > 0.10, respectively, were utilized in the variable selection process. A two-sided *P*-value of < 0.05 was considered statistically significant.

Based on the findings of multiple logistic regression analysis, a prediction model for IKPLAS was developed. By using the RMS package, a nomogram was constructed to evaluate the risk of invasive syndrome in patients with liver abscesses. The agreement between the observed and predicted results was assessed with the Hosmer–Lemeshow (H–L) goodness-of-fit test. The glmnet and ROCR packages in the R program were used to plot the receiver operating characteristic (ROC) curves for each model. Bootstrap resampling with 1,000 iterations was performed to validate the model, and the area under the ROC (AUC) value was computed as a measure of discrimination. The validation cohort was used to validate the model, and the AUC value and calibration curves were utilized to evaluate the discriminative ability and predictive accuracy of the model, respectively. The net clinical benefit of the model was evaluated based on decision curve analysis (DCA).

## 3 Results

### 3.1 Baseline characteristics

**Figure 1:**
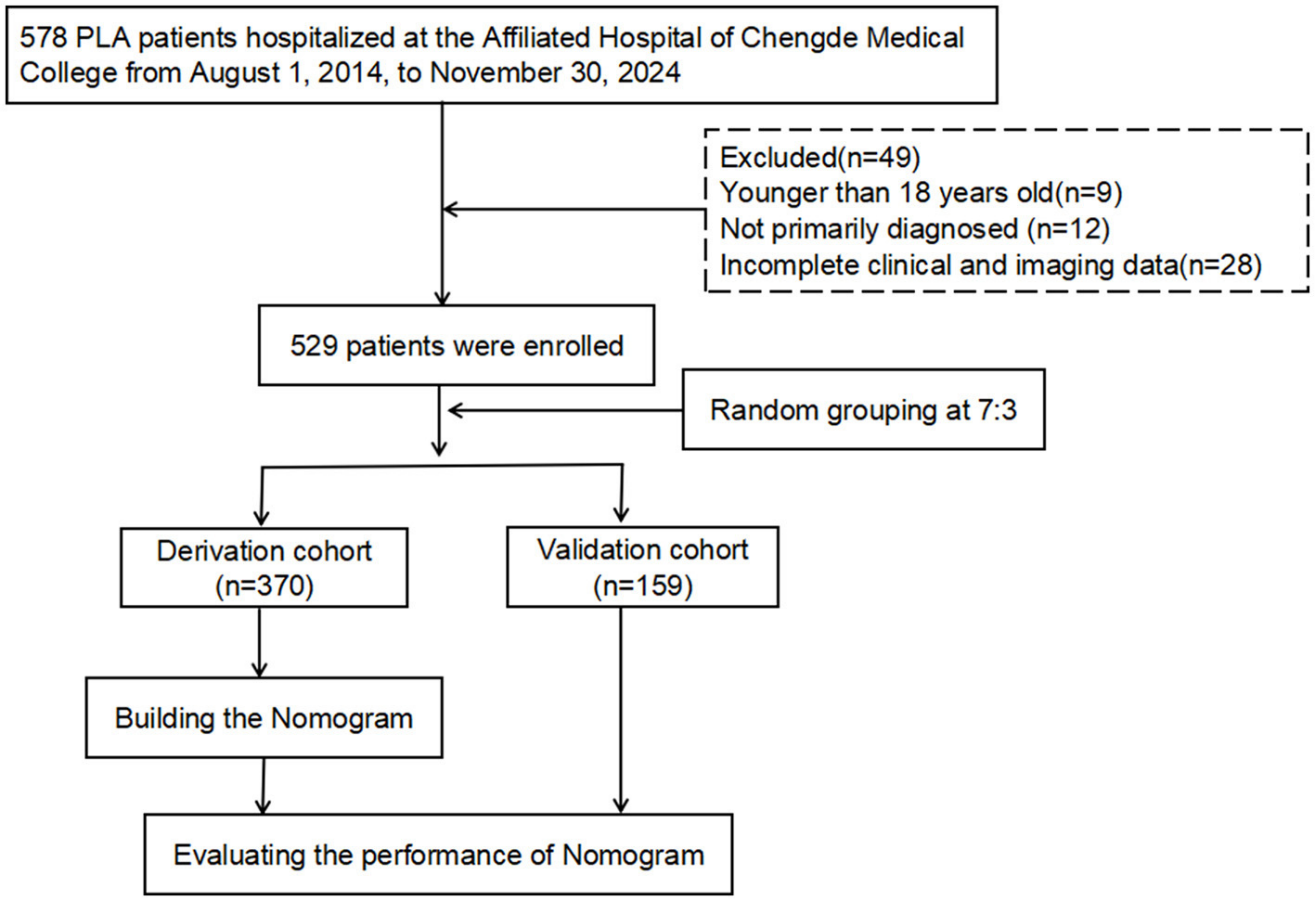
Flowchart of the study design.

Based on the inclusion-exclusion criteria screening process, this study enrolled 529 patients with KPLA, including 322 males and 207 females (Figure 1). Thirty-three patients (6.2%) developed invasive syndrome (designated as the IKPLAS group), while the remaining 496 patients with non-invasive syndrome constituted the NIKPLAS group. In the IKPLAS group, one patient presented with concurrent pulmonary abscess and endophthalmitis; other metastatic infections manifested as solitary conditions: endophthalmitis (*n* = 5), cerebral abscess (*n* = 2), pulmonary abscess (*n* = 14), subphrenic abscess (*n* = 3), intra-abdominal abscess (*n* = 3), and chest wall abscess (*n* = 5). The baseline demographic characteristics and clinical parameters of both groups are detailed in Table 1.

**Table 1:** Comparison of baseline characteristics and clinical data between patients with NIKPLAS and IKPLAS.

**Variables**	**NIKPLAS (*n* = 496)**	**IKPLAS (*n* = 33)**	***t*/*Z*/χ^2^**	***P***
**General information**				
Age	59.4 ± 13.5	59.3 ± 9.8	0.039	0.969
**Gender**				
Male	302 (57.1)	20 (3.8)	0.001	0.974
Female	194 (36.7)	13 (2.5)		
**Comorbidities**				
Viral hepatitis	15 (2.8)	13 (2.5)	81.646	< 0.001
Biliary disease	106 (20.0)	1 (0.2)	6.450	0.035
Malignant neoplasm	52 (9.8)	1 (0.2)	1.914	0.167
Hypertension	151 (28.5)	9 (1.7)	0.147	0.701
T2DM	174 (32.9)	21 (4.0)	10.841	0.002
Hyperuricemia	25 (4.7)	4 (0.8)	2.994	0.084
**Clinical features**				
Temperature (°C)	38.7 ± 2.3	39.1 ± 1.2	−0.924	0.356
Shiver	258 (48.8)	17 (3.2)	0.003	0.956
Poor appetite	259 (49.0)	15 (2.8)	0.567	0.452
Vomiting	428 (80.9)	24 (4.5)	4.577	0.038
Abdominal pain	123 (23.3)	10 (1.9)	0.498	0.480
Pleural effusion	111 (21.0)	10 (1.9)	1.101	0.294
Ascites	22 (4.2)	2 (0.4)	0.189	0.664
Pulmonary infection	54 (10.2)	10 (1.9)	10.968	0.002
Perihilar abscess	6 (1.1)	1 (0.2)	0.785	0.375
Septic shock	49 (9.3)	10 (1.9)	13.025	< 0.001
MODS	20 (3.8)	2 (0.4)	0.319	0.572
**Laboratory parameters**				
WBC ( × 10^9^)	9.3 (6.6, 12.7)	10.1 (6.8, 14.6)		0.409
Neutrophil ratio (%)	78.5 (69.2, 86.8)	77.5 (66.4, 85.6)		0.592
PLT ( × 10^9^)	230 (130.5, 333.5)	216 (77.0, 289.0)		0.251
Hb (g/L)	115.1 ± 20.2	109.4 ± 19.7	1.573	0.116
CRP (mg/L)	106.0 (61.0, 163.8)	125.4 (84.7, 222.7)		0.046
Blood glucose (mmol/L)	7.1 (5.7, 10.9)	8.4 (6.3, 12.8)		0.115
ALP (U/L)	153 (112.7, 214.6)	149.5 (103, 250.5)		0.976
International normalized ratio	1.14 ± 0.18	1.10 ± 0.13	1.156	0.248
Fibrinogen (g/L)	5.6 (4.6, 6.5)	5.6 (4.5, 7.8)		0.505
**Imaging findings**				
Abscess location			0.485	0.922
Left lobe	147 (27.8)	10 (1.9)		
Right lobe	272 (51.4)	17 (3.2)		
Left and right lobe	74 (14.0)	6 (1.1)		
Caudate lobe	3 (0.6)	0 (0)		
Number of abscesses			0.413	0.813
Single	275 (52.0)	20 (3.8)		
Multiple	221 (41.8)	13 (2.5)		
Abscess diameter	59 (42.8, 78.0)	51 (36.0, 65.0)		0.025
Gas-containing abscess	31 (5.9)	19 (3.6)	95.239	< 0.001
**Treatments**			0.475	0.789
Antibiotics	208 (39.3)	12 (2.3)		
Antibiotics and drainage	287 (54.3)	21 (4.0)		
Operative drainage	1 (0.2)	0 (0)		
SOFA score	3.0 (2.0, 5.0)	5.0 (4.0, 7.0)		< 0.001
**Metastatic Infection Sites**				
Eye	5		
Brain	2		
Lung	14		
Subphrenic	3		
Abdominal Cavity	3		
Chest wall	5		
Eye and Lung	1		

### 3.2 Multivariate logistic regression analysis for metastatic syndrome in patients with KPLA

In the univariate analysis, the factors showing a significant association with the development of invasive syndrome in patients with KPLA were viral hepatitis (*P* < 0.001), biliary disease (*P* = 0.035), T2DM (*P* = 0.002), vomiting (*P* = 0.038), pulmonary infection (*P* = 0.002), septic shock (*P* < 0.001), CRP level (*P* = 0.046), abscess diameter (*P* = 0.025), presence of a gas-containing abscess (*P* < 0.001), and SOFA score (*P* < 0.001). In the multivariate logistic regression analysis, viral hepatitis (*P* < 0.001), T2DM (*P* < 0.001), abscess diameter (*P* = 0.005), presence of a gas-containing abscess (*P* < 0.001), and SOFA score (*P* = 0.022) were identified as the independent predictors of IKPLAS (Table 2).

**Table 2:** Results of univariate and multivariate logistic regression analyses.

**Variables**	**Univariate analysis**		**Multivariate analysis**	
	**OR (95% CI)**	***P*** **value**	**OR (95% CI)**	***P*** **value**
Viral hepatitis	20.757 (8.722–49.396)	< 0.001	65.319 (14.466–294.936)	< 0.001
Biliary disease	0.116 (0.016–0.857)	0.035	0.098 (0.009–1.016)	0.052
T2DM	3.247 (1.560–6.758)	0.002	10.311 (2.871–37.030)	< 0.001
Vomiting	0.426 (0.190–0.955)	0.038	1.162 (0.329–4.106)	0.816
Pulmonary infection	3.618 (1.633–8.013)	0.002	0.936 (0.238–3.682)	0.924
Septic shock	3.949 (1.776–8.778)	< 0.001	1.055 (0.282–3.945)	0.936
CRP level	1.004 (1.000–1.009)	0.046	1.003 (0.996–1.010)	0.352
Abscess diameter	0.982 (0.967–0.998)	0.025	0.965 (0.942–0.989)	0.005
Gas-containing abscess	20.270 (9.289–44.228)	< 0.001	108.757 (24.100–490.781)	< 0.001
SOFA score	1.311 (1.132–1.518)	< 0.001	1.346 (1.044–1.736)	0.022

### 3.3 Development of a nomogram prediction model for IKPLAS patients

**Figure 2:**
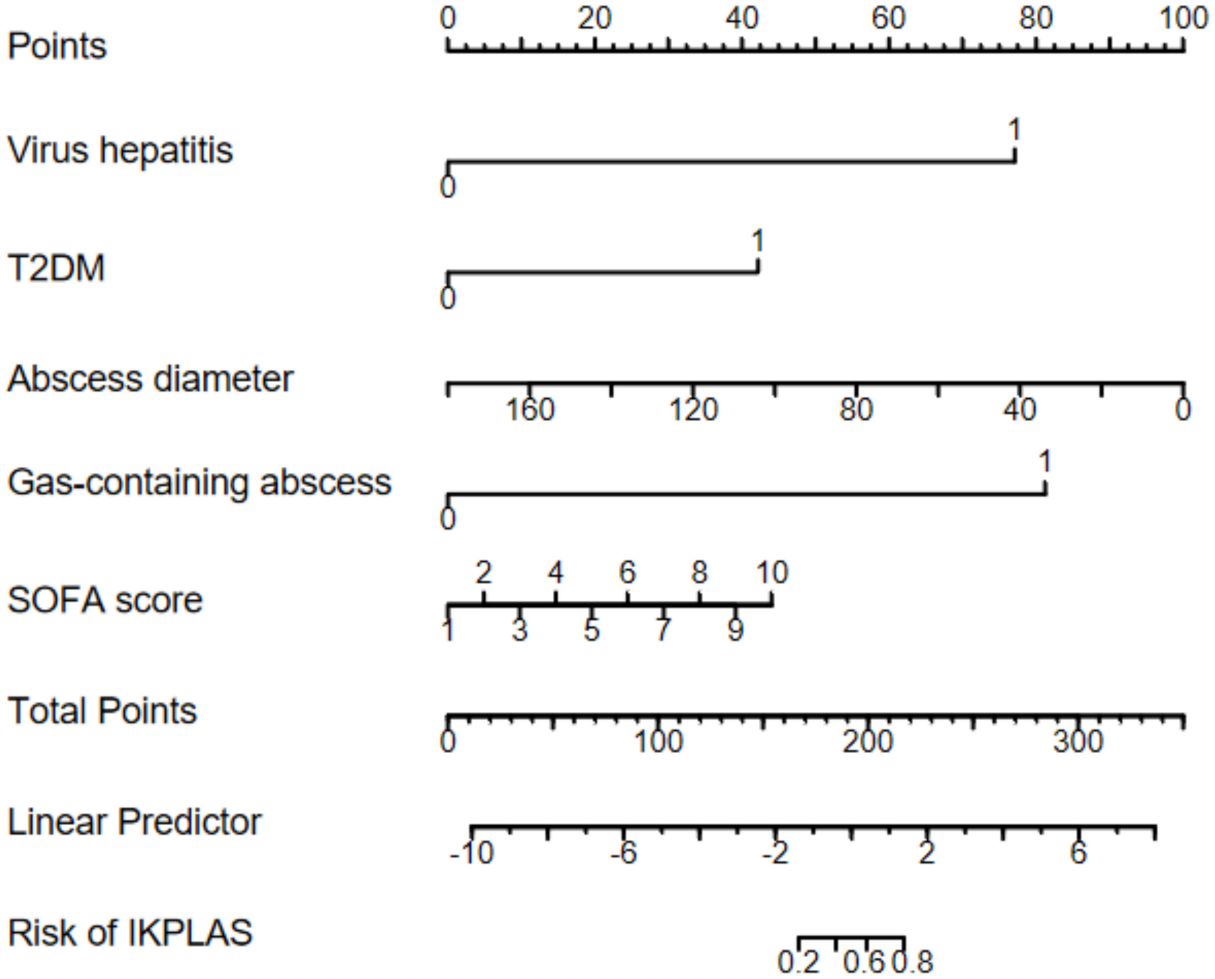
Nomogram for predicting IKPLAS in patients with pyogenic liver abscess.

According to the multivariate logistic regression analysis, viral hepatitis, T2DM, abscess diameter, presence of a gas-containing abscess, and SOFA score were the independent risk factors for invasive syndrome in patients with liver abscess. Based on these predictors, we developed a predictive model and constructed a nomogram (Figure 2) for visual representation. The model exhibited good calibration based on the H–L goodness-of-fit test (χ^2^ = 4.171, *P* = 0.841).

### 3.4 Validation of the nomogram prediction model for IKPLAS patients

**Figure 3:**
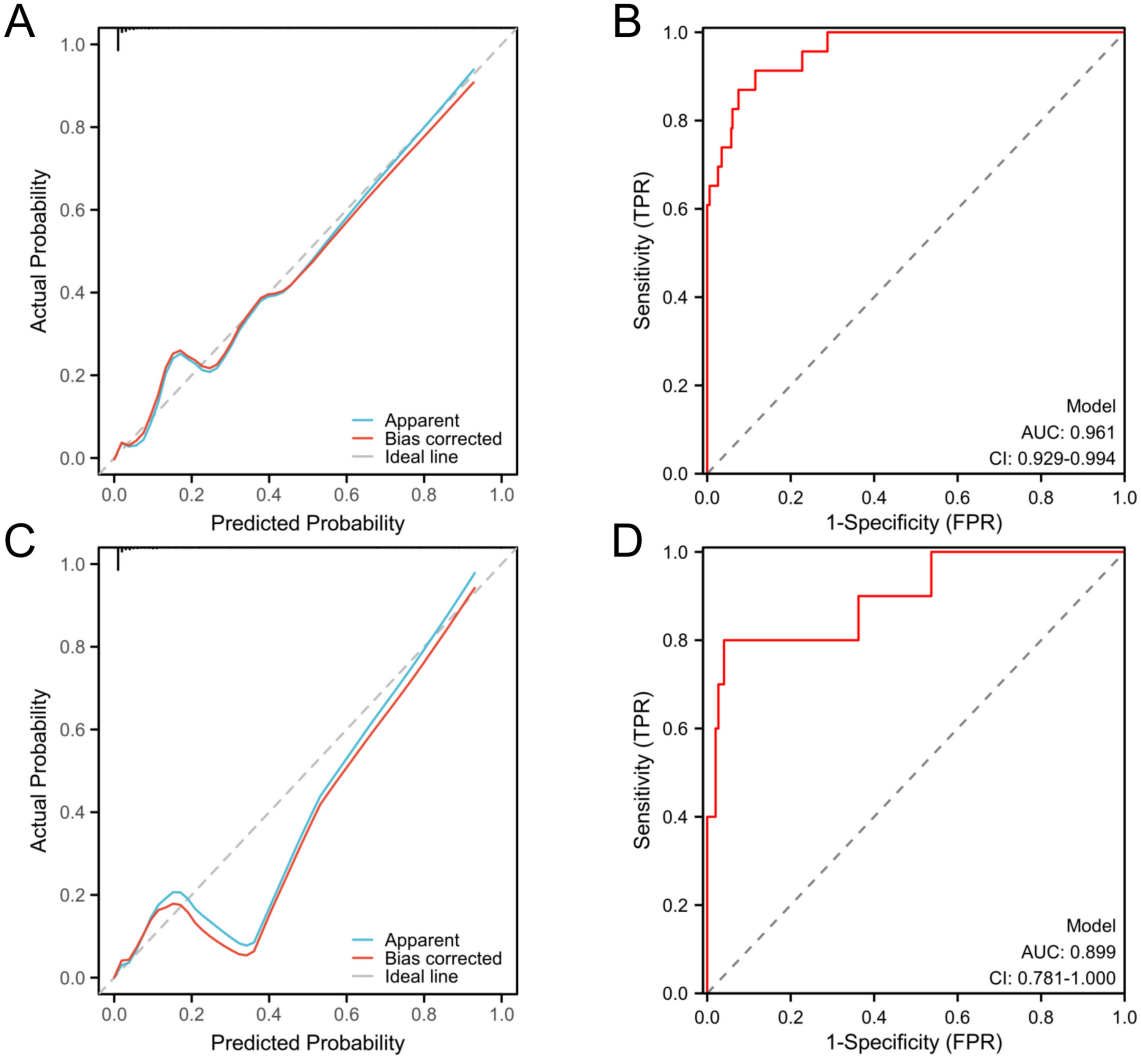
Calibration and discrimination curves of the prognostic nomogram model. Calibration curves **(A)** and discrimination curve **(B)** of the derivation cohort. Calibration curves **(C)** and discrimination curve **(D)** of the validation cohort. ROC, receiver operating characteristic; AUC, area under the ROC curve.

The predictive performance of the nomogram was evaluated by the ROC curve analysis in both derivation and validation cohorts. In the derivation cohort, the model showed excellent discriminative ability with an AUC value of 0.961 (95% CI: 0.929–0.994; Figure 3B). The validation cohort showed robust performance with an AUC value of 0.899 (95% CI: 0.781–1.000; Figure 3D). Calibration curves revealed strong agreement between the predicted and observed outcomes in both derivation (Figure 3A) and validation (Figure 3C) cohorts, which confirmed the clinical reliability of the model.

### 3.5 Clinical utility assessment of the nomogram prediction model for IKPLAS patients

**Figure 4:**
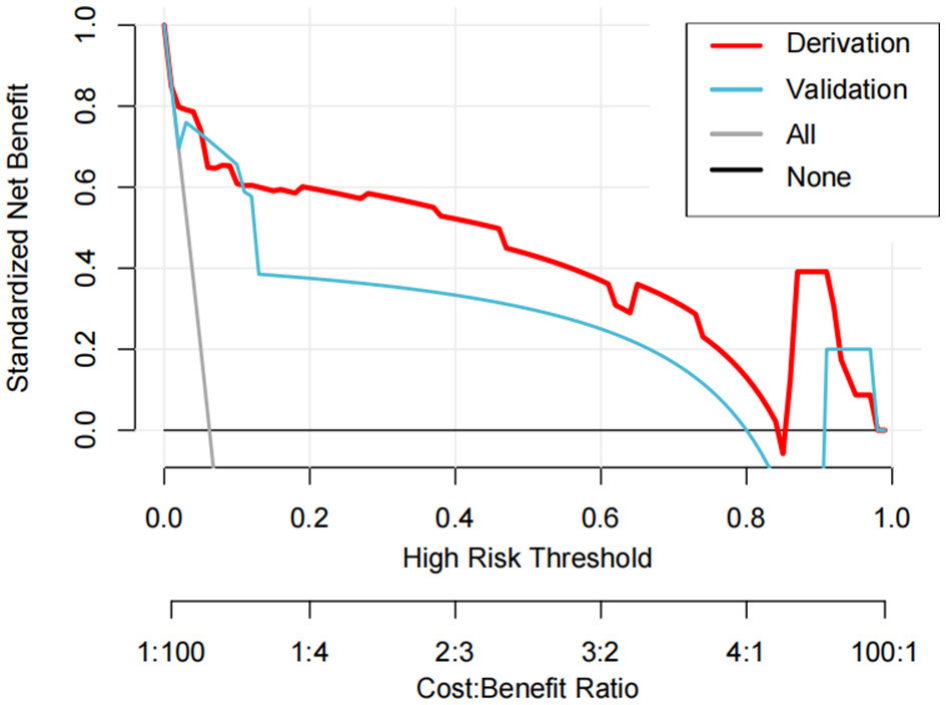
The decision curve analysis curves of the nomogram model in the derivation and validation cohorts.

DCA addresses the limitations of conventional evaluation metrics, which focus solely on predictive accuracy while neglecting clinical utility. In DCA plots, the horizontal line represents the “no-intervention” strategy (net benefit of zero), and the diagonal line denotes the “universal intervention” strategy (assuming treatment for all patients). The findings revealed that the model effectively predicts the risk of invasive syndrome development in patients with KPLA. This conclusion was validated through DCA in both derivation and validation cohorts (Figure 4).

## 4 Discussion

PLA is caused by various pathogenic microorganisms and is characterized by an acute onset and rapid progression. This condition frequently results in hepatic dysfunction and often leads to severe complications with increased mortality risk when left untreated. Notably, IKPLAS, a metastatic complication of KPLA, shows poor prognosis and a high mortality rate, thereby posing considerable clinical threats (13). Although previous studies have identified risk factors associated with invasive syndrome development in patients with KPLA, there is a lack of systematic quantitative assessment tools to accurately predict the likelihood of invasive syndrome occurrence in this population. Our study addresses this gap by establishing a predictive nomogram based on the identified risk factors for IKPLAS in patients with KPLA. This evidence-based tool provides clinicians with quantitative support for early intervention strategies, ultimately improving patient outcomes (14).

Viral hepatitis constitutes a group of liver-targeting infectious diseases caused by hepatotropic viruses (15, 16), with an increasing global incidence in recent decades (17). Through multitargeted pathogenic cascades, including hepatic immune barrier disruption, systemic immunosuppression, gut microbiota translocation, and metabolic dysregulation, viral hepatitis substantially elevates the risk of invasive syndrome in KPLA patients. Chronic viral hepatitis-induced liver injury critically compromises hepatic innate immunity: hepatitis B virus (HBV)/hepatitis C virus (HCV) infection suppresses phagocytic activity and pathogen clearance capacity of Kupffer cells, facilitating intrahepatic colonization and abscess formation by *Klebsiella pneumonia* (18). Virus-mediated apoptosis of sinusoidal endothelial cells disrupts the hepatic vascular barrier, promoting bacterial dissemination through the portal system (19). Concurrently, chronic HBV/HCV infection promotes CD8+ T cell exhaustion, impairing both intracellular pathogen elimination and extracellular bacterial immune surveillance (20). Virus-triggered hyperinflammation exacerbates tissue damage while upregulating the expression of bacterial virulence factors, thereby accelerating hematogenous spread of the bacterial infection (21). Portal hypertension increases intestinal permeability, enabling enteric pathogens to translocate through the gut-liver axis, thus establishing a vicious cycle of secondary infection (22). These synergistic mechanisms collectively predispose patients to the progression of invasive syndrome.

T2DM is recognized as a major risk factor for KPLA, with approximately 40% of KPLA patients presenting with comorbid diabetes (23). Chronic hyperglycemia in T2DM impairs both innate and adaptive immune responses through oxidative stress-mediated suppression of neutrophil chemotaxis, phagocytic activity, and neutrophil extracellular trap (NET) formation, thereby reducing bacterial clearance efficiency (24). Mechanistically, glucose serves as a carbon source to enhance *Klebsiella pneumoniae* virulence by increasing capsular polysaccharide production (e.g., K1/K2 serotypes) and upregulating mucoid phenotype-associated genes through the cAMP-Crp signaling pathway, thereby increasing antiphagocytic capacity (25). Furthermore, hyperglycemia-induced glycocalyx shedding and tight junction disruption in vascular endothelia facilitate bacterial penetration across vascular barriers, promoting metastatic infections (26). T2DM establishes a self-reinforcing pathogenic cycle through immunosuppression, metabolic microenvironment remodeling, and vascular injury; these conditions collectively exacerbate the invasive syndrome risk in KPLA patients. Consequently, the early detection and management of liver abscesses are critical in diabetic patients.

Contrary to conventional understanding that a larger abscess size is associated with severe local inflammation, our nomogram paradoxically revealed an inverse correlation of abscess diameter with invasive syndrome risk. This phenomenon reflects synergistic interactions among hypervirulent pathogens, host immunometabolic dysregulation, and delayed diagnosis. HvKP strains with elevated capsular polysaccharide production and upregulated mucoid phenotype-associated genes can form microabscesses during the early infection stage. The capsular components evade immune clearance by suppressing complement activation and NET formation, thereby facilitating hematogenous dissemination (27). In diabetic or immunocompromised hosts, regulatory T cells exacerbate dissemination risks through IL-10-mediated suppression of Th17-dependent neutrophil recruitment, thereby impairing abscess containment (28). Furthermore, the absence of classical symptoms (e.g., high fever and right upper quadrant pain) in microabscess patients often delays medical intervention until bacteremia or metastatic infections develop (29).

Intralesional gas formation is a critical biomarker of hvKP pathogenicity, reflecting dynamic host-pathogen metabolic interactions and immune dysregulation. Gas-mediated dissemination occurs through two synergistic pathways: mechanical stress and hypoxic metabolic adaptation. Elevated gas pressure increases abscess wall tension, disrupting hepatic sinusoidal endothelial tight junctions (ZO-1 and claudin-5) to create physical conduits for bacterial vascular invasion (30). Concurrently, gas production correlates with localized hypoxia, which activates the fumarate and nitrate reductase regulator to upregulate hvKP invasion-associated genes (cf29a and ybtA), thereby enhancing endothelial adhesion and transmigration (31). Notably, 30% of gas-forming abscesses show polymicrobial infection with Escherichia coli or anaerobe bacteria, where β-lactamase production and superantigens potentiate hvKP antibiotic resistance and invasiveness (32). Gas-forming pyogenic liver abscess (GFPLA) exhibits distinct multimodality imaging features. On ultrasonography, intralesional gas appears as scattered, linear, or clustered hyperechoic foci. This phenomenon originates from repeated sound wave reflection at gas–tissue interfaces, which produces multiple linear reverberation artifacts posterior to the gas collection location (termed the “ring-down” or “comet-tail” sign). CT reveals hypoattenuating gas pockets with approximately−1000 Hounsfield units (HU), which is markedly distinct from both purulent material (−10 to 30 HU) and hepatic parenchyma (40–70 HU). MRI exhibits signal voids on both T1- and T2-weighted sequences because of gas-induced magnetic susceptibility artifacts, which are accentuated on gradient-echo sequences. While no formal diagnostic criteria exist for GFPLA, its characteristic ultrasonographic, CT, and MRI features enable conclusive radiographic identification (33). Therefore, the presence of gas within a liver abscess is a marker of an increased risk of developing the invasive syndrome.

SOFA score is a validated metric for quantifying organ dysfunction/failure in critically ill patients (34). In the present study, an elevated SOFA score was established as an independent risk factor for IKPLAS patients. First, sepsis-associated MODS reflected by high SOFA scores (e.g., respiratory failure and coagulopathy) synergizes with systemic inflammation; *Klebsiella pneumoniae*-derived capsular polysaccharides and lipopolysaccharides activate the TLR4/NF-κB pathway, triggering a cytokine storm (IL-6 and TNF-α) that disrupts vascular endothelial barriers, thereby facilitating hematogenous dissemination of the pathogen to distant organs (35). Second, hypervirulent hvKP strains establish a self-perpetuating cycle of organ invasion and immune exhaustion; subsequently, K1/K2 serotypes utilize adhesins to breach vascular endothelia and establish metastatic infections (36). Third, metabolic disturbances, such as lactate accumulation and iron overload, enable hvKP to adapt and proliferate under hypoxic conditions (37). These findings position the SOFA score not only as an organ failure quantifier but also as a composite biomarker of systemic “immune-metabolic-microbial” dysregulation, whose integration into predictive models significantly enhances early warning capacity for invasive syndrome.

Based on the above five independent risk factors, this study constructed a concise and intuitive nomogram. We acknowledge that factors such as the Charlson Comorbidity Index (CCI), dietary habits, and alcohol consumption may be related to the progression of KPLA and the occurrence of invasive syndrome. These factors were not included in the nomogram of this study mainly due to the following two reasons: First, in our retrospective cohort, the complete data on patients' dietary habits and alcohol consumption, as well as the records of chronic comorbidities in some patients, are incomplete, and their inclusion may introduce selection bias; Second, this study focuses on predicting the acute progression of invasive syndrome in KPLA patients, while CCI is used to quantify previous chronic comorbidities. Although these factors were not included in this study, we recognize their potential value and emphasize that future prospective studies with larger sample sizes and more comprehensive data collection should be conducted to further explore the association between these additional factors and invasive KPLA. Previous studies have shown an association between elevated CRP and reduced hemoglobin levels and IKPLAS occurrence (38, 39). However, these variables did not exhibit independent prognostic significance in our cohort. An elevated CRP level, although indicative of systemic inflammation, shows limited specificity in differentiating infectious etiologies or disease progression stages in patients with pyogenic liver abscess. Moreover, confounding effects of comorbidities (diabetes mellitus) and therapeutic interventions (antibiotic administration) may obscure causal associations between CRP levels and invasive syndrome outcomes. A reduced hemoglobin level (< 90 g/L) exhibited strong collinearity with diabetic microangiopathy and chronic kidney disease in multivariate models, suggesting that anemia is potentially a secondary manifestation of diabetes/renal impairment rather than an independent driver of invasive progression (40).

The global incidence of *Klebsiella pneumoniae*-associated liver abscess (KPLA) has risen significantly. Distinguishing community-acquired (CA-KPLA) and healthcare-associated (HA-KPLA) types is critical for understanding epidemiology and clinical implications. Epidemiologically, CA-KPLA accounts for 60%−75% of KPLA cases in Asia and 40%−60% in Western countries, mainly caused by hypervirulent *K. pneumoniae* (hvKP, especially K1/K2 serotypes). These strains asymptomatically colonize the gut and spread via fecal-oral routes or close contact in communities. Clinically, CA-KPLA affects middle-aged patients with diabetes and is more likely to progress to invasive syndromes (e.g., endophthalmitis) due to high hvKP virulence. HA-KPLA represents 25%−40% of global KPLA cases, more prevalent in tertiary hospitals. It is mainly caused by classical *K. pneumoniae* (cKP), mostly multidrug-resistant (MDR) or carbapenem-resistant (CRKP), linked to broad-spectrum antibiotic use in healthcare. HA-KPLA spreads via contaminated devices, healthcare workers' hands, or invasive procedures. Patients have severe comorbidities or long hospital stays; though with lower metastatic infection rates than CA-KPLA, they face higher mortality due to MDR/CRKP. This difference highlights the need for targeted risk assessment, aligning with our study's goal of developing an invasive KPLA nomogram (41).

Beyond transmission scenarios, KPLA shows distinct age and gender patterns. Epidemiologically, it mainly affects middle-aged and elderly people: global data notes peak incidence in those aged 50–70, accounting for 60%−75% of cases. This age bias ties to age-related changes (e.g., declining liver function) and more comorbidities (diabetes, biliary diseases), weakening resistance to *K. pneumoniae*. HA-KPLA patients are older than CA-KPLA ones, likely due to the elderly's more frequent healthcare contact and severe comorbidities (malignancy, post-transplant) (42). In gender, global KPLA studies consistently show male predominance: 55%−70% are male, with a 1.2:1 to 2:1 male-to-female ratio. Potential causes include hormones and lifestyle factors. Notably, male predominance is more obvious in CA-KPLA (up to 70%), possibly linked to higher uncontrolled diabetes rates in males—diabetes is key for hvKP-induced liver abscesses. These traits reflect KPLA's epidemiology, hint at targeted prevention for specific groups, and align with this study's focus on invasive KPLA risk stratification.

The antibiotic susceptibility profiles of clinical hvKP strains isolated from PLA patients have important clinical reference value: globally, 80% to 90% of community-acquired (CA)-hvKP strains remain sensitive to broad-spectrum β-lactam antibiotics (such as ceftriaxone and cefotaxime), and the resistance rates to fluoroquinolones (levofloxacin) and aminoglycosides (gentamicin) are usually < 15%, which provides a basis for empirical medication for CA-PLA caused by suspected hvKP. However, the rate of multidrug resistance (MDR) in healthcare-associated (HA)-hvKP strains is on the rise: in high-burden areas, 20% to 35% of the strains produce extended-spectrum β-lactamases (ESBLs) and are resistant to cephalosporins; 5% to 10% are carbapenem-resistant hvKP (CR-hvKP). Such drug resistance limits treatment options and highlights the necessity of promptly conducting antibiotic susceptibility testing to guide targeted therapy—this is particularly critical for invasive cases, as delayed effective treatment will exacerbate poor prognosis (43).

The results of DCA revealed superior clinical utility of the developed nomogram when the risk probability threshold exceeded 20%. Patients with a score exceeding the nomogram score cutoff of 170 points would benefit from therapeutic escalation, such as antibiotic treatment, percutaneous drainage, or transfer to the intensive care unit. This would prevent the development of IKPLAS, optimizing resource allocation and maintaining clinical efficacy.

This study has several limitations. First, the retrospective design introduces potential selection bias inherent to observational studies. Second, key parameters, including procalcitonin, interleukin, and blood urea nitrogen levels, were frequently missed during the initial patient triage, leading to their exclusion from our analysis; hence, future investigations should explore the prognostic potential of these parameters for invasive syndrome. Third, although internal validation was performed, the single-center derivation cohort requires external validation across diverse populations to strengthen the clinical generalizability of the nomogram. Fourth, we did not include the metagenomic next-generation sequencing (mNGS) data of the strains and their association with IKPLAS. In the future, there is still room for further research in exploring the genetic factors related to IKPLAS.

## 5 Conclusion

We developed and validated a predictive model that shows excellent discriminative performance for stratifying invasive syndrome risk in patients with liver abscess. The constructed nomogram incorporates five independent predictors, namely viral hepatitis, T2DM, abscess diameter, presence of a gas-containing abscess, and SOFA score, achieving balanced sensitivity and specificity. This tool can enable clinicians to (1) personalize antimicrobial regimens based on individual risk profiles, (2) prioritize percutaneous drainage for high-risk cases, and (3) optimize healthcare resource allocation, thereby reducing both incidence and mortality rate of invasive syndrome.

## Data Availability

The raw data supporting the conclusions of this article will be made available by the authors, without undue reservation.
